# Advancing Diabetes Research: A Novel Islet Isolation Method from Living Donors

**DOI:** 10.3390/ijms25115936

**Published:** 2024-05-29

**Authors:** Eleonora Di Piazza, Laura Todi, Gianfranco Di Giuseppe, Laura Soldovieri, Gea Ciccarelli, Michela Brunetti, Giuseppe Quero, Sergio Alfieri, Vincenzo Tondolo, Alfredo Pontecorvi, Antonio Gasbarrini, Enrico Celestino Nista, Andrea Giaccari, Giovambattista Pani, Teresa Mezza

**Affiliations:** 1 Endocrinology and Diabetology Unit, Fondazione Policlinico Universitario Gemelli IRCCS, 00168 Roma, Italy; 2 Department of Medicine and Translational Surgery, General Pathology Section, Università Cattolica del Sacro Cuore, 00168 Roma, Italy; 3 Digestive Surgery Unit, Fondazione Policlinico Universitario Gemelli IRCCS, 00168 Roma, Italy; 4 Digestive Surgery Unit, Ospedale Isola Tiberina—Gemelli Isola, 00186 Roma, Italy; 5 Pancreas Unit, CEMAD Centro Malattie dell’Apparato Digerente, Medicina Interna e Gastroenterologia, Fondazione Policlinico Universitario Gemelli IRCCS, 00168 Roma, Italy

**Keywords:** pancreatic islets, type 2 diabetes, islet isolation, insulin secretion

## Abstract

Pancreatic islet isolation is critical for type 2 diabetes research. Although -omics approaches have shed light on islet molecular profiles, inconsistencies persist; on the other hand, functional studies are essential, but they require reliable and standardized isolation methods. Here, we propose a simplified protocol applied to very small-sized samples collected from partially pancreatectomized living donors. Islet isolation was performed by digesting tissue specimens collected during surgery within a collagenase P solution, followed by a Lympholyte density gradient separation; finally, functional assays and staining with dithizone were carried out. Isolated pancreatic islets exhibited functional responses to glucose and arginine stimulation mirroring donors’ metabolic profiles, with insulin secretion significantly decreasing in diabetic islets compared to non-diabetic islets; conversely, proinsulin secretion showed an increasing trend from non-diabetic to diabetic islets. This novel islet isolation method from living patients undergoing partial pancreatectomy offers a valuable opportunity for targeted study of islet physiology, with the primary advantage of being time-effective and successfully preserving islet viability and functionality. It enables the generation of islet preparations that closely reflect donors’ clinical profiles, simplifying the isolation process and eliminating the need for a Ricordi chamber. Thus, this method holds promises for advancing our understanding of diabetes and for new personalized pharmacological approaches.

## 1. Introduction

Pancreatic islet isolation is a critical procedure in diabetes research and is of great importance for studying the pathophysiology of islet cells. Particularly, type 2 diabetes (T2D) has proven to be highly heterogeneous both in its clinical and molecular features [1], hence resulting in the lack of a comprehensive understanding of its underlying causes. Even though the -omics approaches provide a thorough view of the islets’ molecular footprints in health and disease [2,3,4], the results are not always matching [5], and consequently, a unique profiling of diabetes trajectory from its very beginning is still to be achieved. Furthermore, transcriptional and proteomic analyses—although very informative from the molecular side—cannot replace functional studies. Thus, developing a reliable and, ideally, standardized islet isolation method represents a crucial issue in the field of diabetes, both for a deeper understanding of the cellular and molecular basis of the disease and for clinical/therapeutic purposes [6,7,8].

Several protocols have been developed and improved over the years from the standardized Ricordi chamber method designed for human pancreata digestion [9,10]: the attention has been focused not only on samples from human derivation [11,12,13,14,15] but also on various preclinical models such as rodents [16,17,18,19] and pigs [20,21,22,23]. Indeed, rodents have been variously used to model the islet pathophysiology in health and disease, despite the metabolic [24] and structural [25] differences with human islets; on the other hand, there is a strong concern about pig islets as a promising therapeutic resource for xenotransplantation in type 1 diabetes treatment [26,27].

For research purposes, isolated human islets mostly derive from deceased organ donors [28,29,30,31,32,33], with the main advantage of having considerable starting material, standardized conditions, and an overall high purification yield. On the other hand, clinical information and family history about brain-dead organ donors are limited [34], resulting in poorly characterized samples and the lack of clinical in vivo features to be correlated with functional or molecular assays.

In this work, we provide a novel method for isolating pancreatic islets from living patients undergoing a partial pancreatectomy. All individuals underwent a deep metabolic evaluation and functional studies before the surgery, thus allowing for distinguishing different metabolic conditions and correlating any in vitro features revealed on the pancreas samples to a specific in vivo profile. The main advantage of this procedure is that we finally obtain samples enriched with living pancreatic islets that reflect the clinical profile of their donors, starting from a minimal amount (1–1.5 g) of healthy tissue. Lastly, we do not use a Ricordi chamber, which considerably simplifies the whole procedure in terms of time and cost-effectiveness.

## 2. Results

### 2.1. Patient Characterization and Surgical Procedure

#### Study Design and Experimental Procedures

Patient characterization and surgical procedures were performed as previously described [35,36,37,38]. Patients scheduled for pylorus-preserving pancreatoduodenectomy were recruited at the Digestive Surgery Unit and studied at the Centre for Endocrine and Metabolic Diseases Unit, Agostino Gemelli University Hospital, Rome, Italy. Indications for surgery were periampullary tumors, pancreatic intraductal papillary tumors, mucinous cystic neoplasm of the pancreas, and non-functional pancreatic neuroendocrine tumors.

Each subject underwent an oral glucose tolerance test, a hyperinsulinemic–euglycemic clamp, a hyperglycemic clamp, and a mixed-meal test 1 week before the surgical procedure (Table 1).

- Oral Glucose Tolerance Test:

Normal glucose metabolism and glucose tolerance status were determined by a standard 75 g oral glucose tolerance test measuring glycemia, insulin, and C-peptide at 0, 30, 60, 90, 120 min after the glucose load.

- Hyperinsulinemic–Euglycemic Clamp Procedure:

The hyperinsulinemic–euglycemic clamp test was performed after a 12 h overnight fast using insulin 40 mIU⋅m^−2^⋅min^−1^ of body surface, according to DeFronzo et al. [39]. A primed constant infusion of insulin was administered (Actrapid HM, 40 mIU⋅m^−2^⋅min^−1^; Novo Nordisk, Copenhagen, Denmark). The constant rate for the insulin infusion was reached within 10 min to achieve steady-state insulin levels. In the meantime, a variable infusion of 20% glucose was started with a separate infusion pump, and the rate was adjusted on the basis of plasma glucose samples drawn every 5 min to maintain the plasma glucose concentration at each participant’s fasting plasma glucose level. During the last 20 min of the clamp procedure, plasma samples from blood drawn at 5 to 10 min intervals were used to determine glucose and insulin concentrations. Whole-body peripheral glucose utilization was calculated during the last 30 min period of the steady-state insulin infusion and was measured as the mean glucose infusion rate (mg⋅kg^−1^⋅min^−1^).

- Hyperglycemic Clamp Procedure:

The plasma glucose was clamped at a stable level of 125 mg/dL above the fasting blood glucose concentration. The hyperglycemic clamp was started with a 200 mg/mL bolus dose of dextrose (150 mg/kg) administered into the antecubital vein. Blood was drawn from a cannulated dorsal hand vein on the opposite arm. Venous plasma glucose was analyzed every 5 min with a glucose analyzer, and the infusion of 20% glucose was adjusted to achieve a stable glucose level of 125 mg/dL above the fasting value. Serum samples for insulin and C-peptide were drawn at 0, 2.5, 5, 7.5, 10, 15, 30, 60, 90, 120, 130, 140, and 150 min.

The first-phase insulin release, reflecting the early insulin peak secreted from the pancreatic β-cell in response to glucose stimulation, was calculated as the area under the curve (AUC) during the first 10 min of the clamp by using the trapezium rule. The second-phase insulin release, reflecting β-cell function under sustained elevated glucose levels, was calculated as the AUC from 10 to 120 min. Subsequently, a 5 g arginine bolus was administered to measure maximum C-peptide secretory capacity at a steady-state blood glucose concentration of 250 mg/dL. Combined hyperglycemia- and arginine-stimulated β-cell secretory capacity was calculated as the insulin AUC during the 30 min after the arginine bolus. Insulin and C-peptide AUC values are shown in Table 1 and in Supplementary Figure S1.

- Mixed-Meal Test:

Patients were instructed to consume a meal of 830 kcal (107 kcal from protein, 353 kcal from fat, and 360 kcal from carbohydrates) within 15 min. Blood samples were drawn twice in the fasting state and at 30 min intervals over the following 240 min (sample time 0, 30, 60, 90, 120, 150, 180, 210, and 240 min) for the measurement of plasma glucose, insulin, and C-peptide. Plasma glucose concentrations were determined by the glucose oxidase technique using a glucose analyzer (Beckman Instruments, Palo Alto, CA, USA). Plasma C-peptide was measured by AutoDELPHIA automatic fluoroimmunoassay (Wallac, Turku, Finland), with a detection limit of 17 pmol/L.

### 2.2. Surgical Procedures

Pancreatoduodenectomy was performed according to the pylorus-preserving technique [36]. Briefly, the pancreatic head, the entire duodenum, the common bile duct, and the gallbladder were removed en bloc, leaving a functioning pylorus intact at the gastric outlet. All adjacent lymph nodes were carefully removed. The continuity of the gastrointestinal tract was restored by an end-to-side invaginated pancreatojejunostomy. Further downstream, an end-to-side hepaticojejunostomy and side-to-side gastroenterostomy or an end-to-side pylorus–jejunostomy was made. A pancreas sample was collected during the surgery from the downstream edge of the surgical cut.

### 2.3. Samples Collection and Tissue Digestion

Small pancreatic tissue specimens, sized about 1 cm^3^, are collected in a 50 mL tube during the surgery and immediately soaked in 10–15 mL of cold physiological solution (NaCl 0.9%); to prevent tissue autolysis, the samples should be kept on ice or at +4 °C and the processing time should be minimized as much as possible (longer pancreas ischemia time has been associated to lower islet isolation yields [40]).

The specimens are transferred under a laminar flow hood, and all the further steps are performed in sterile working conditions. Before starting, a 50 mL tube with an adequate volume of pre-warmed Hank’s Balanced Salts Solution (HBSS) should be prepared: usually, a tissue sample of about 1–1.5 g of weight is digested in a final volume of 10 mL, comprising 8 mL HBSS + 2 mL of collagenase P (1.5 U/mg, used at a working concentration of 1.4 mg/mL). If more tissue is available, the volume of HBSS + collagenase should be adjusted accordingly.

The samples are minced into smaller pieces of 1–2 mm^3^ within a 60 × 15 mm petri dish, using tweezers and a surgical blade. The surrounding fat and embedding matrix residues are discarded since their enzymatic digestion creates an oily layer at the top of the final suspension that might interfere with the following isolation steps. Chopped tissue pieces are finally transferred into the tube containing the collagenase P solution in HBSS, and the entire mixture is vigorously hand-shaken. Enzymatic digestion is carried out at 37 °C in a shaking bath for 1 h, pipetting up and down or manually shaking the tube 2–3 times during the whole procedure to ensure the proper mixing and resuspension of the mixture.

### 2.4. Islets Isolation: Filtration and Density Gradient

Before proceeding with the isolation, the following two solutions have to be prepared: HBSS-FBS (90% HBSS–10% FBS (Fetal Bovine Serum)) and 80% Lympholyte (80% Lympholyte–20% HBSS-FBS). Lympholytes 100% and 80% are used at room temperature to create a density separation gradient. HBSS-FBS should be ice-cold to preserve islet vitality as much as possible; FBS is added to sustain cellular recovery.

After the end of the incubation period, the digestion mixture should look homogeneous and turbid, with released cells floating within the suspension and the residual undigested tissue at the bottom of the tube (optimal-quality pancreatic specimens are compact and tend to sediment). The tubes are removed from the water bath and cleaned with 70% ethanol on the external surface, then placed on ice to prevent over-digestion and moved again to sterile working conditions. Meanwhile, a centrifuge should be pre-chilled at 4 °C. The size range for human pancreatic islets is about 50–400 μm, with an average diameter of about 150 μm [41,42,43]. To retain the highest possible number of islets on the upper face of the mesh, we filter the cell suspension using cell strainers of two different sizes (100 μm and 40 μm mesh).

Before proceeding to the serial filtrations, a 100 μm mesh cell strainer is placed over a clean 50 mL tube. About 8–9 mL of supernatant from the digestion mixture (this is the largest volume that can be recollected without picking up tissue residues) is drawn with a serological pipette and gently passed through the 100 μm strainer. The strainer is then enclosed within a clean 60 × 15 mm petri dish, and 1–2 mL of ice-cold HBSS-FBS are rapidly added over the mesh with a p1000 micropipette to avoid air-drying. The process is repeated identically, passing the filtered suspension through a 40 μm mesh cell strainer. Both the upper and the reverse sides of the meshes are thoroughly washed within their respective plates using abundant ice-cold HBSS-FBS. Every 1–2 mL used, all the medium, enriched with pancreatic cells, is accurately recollected from the strainer mesh and from the plate with a p1000 pipette, then put in a 15 mL tube. Even the dish surface has to be washed a couple of times to minimize the loss of the cellular material due to plastic adhesion.

After recollecting the cells in about 14–15 mL of total medium, the tube is centrifuged at 1800 rpm for 5 min at +4 °C. In the meantime, a clean 15 mL tube containing 3 mL of pure Lympholyte should be gently overlaid with 3 mL of 80% Lympholyte, reclining the tube and pouring the medium over its wall. At the end of centrifugation, the supernatant is discarded, while the pellet is resuspended in cold HBSS in two steps (2 + 1 mL) to recover all the material and slowly stratified over the 80% Lympholyte. The tube is centrifuged at 1800 rpm × 10 min at +4 °C, with reduced acceleration/deceleration (or removing the centrifuge brake).

Lympholyte is a cell separation medium commonly used for lymphocyte isolation [44,45,46]. We use this reagent at two different percentages (100% and 80%) so as to create a density gradient of three layers (with denser Lympholyte at the bottom and HBSS at the top) and two interfaces. This procedure allows for a better separation of tissue debris and single cells and the achievement of a final cell pellet highly enriched in pancreatic islets. 

**Figure 1:**
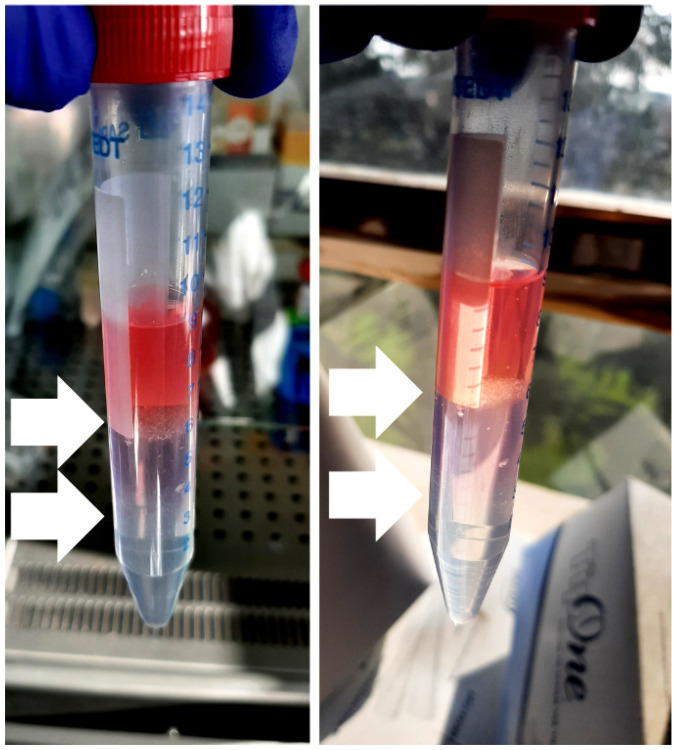
Density separation gradient. Representative pictures of the three-phase density gradient created by layering the HBSS-resuspended sample over two different percentages of Lympholyte (80% HBSS and 100%) from the top to the bottom of the tube. The white arrows indicate the floating interphases (at 6 and 3 mL, approximately) enriched with pancreatic islets; the lower interphase is barely visible.

**Figure 2:**
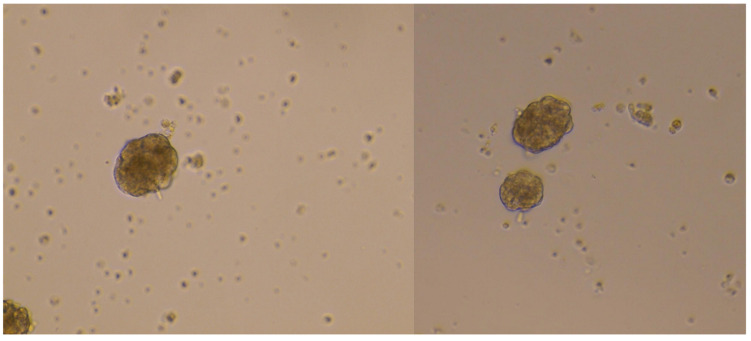
Brightfield images of purified human pancreatic islets after the isolation and a recovery period: 20× enlargements, acquired through EVOS™ XL Core Imaging System (#AMEX1000, Thermo Fisher Scientific, Waltham, MA, USA).

Upon centrifugation, islets form two floating rings at the two interfaces (the superior one at approximately 6 mL and the lower one at approximately 3 mL—the latter sometimes barely visible if the starting material is scarce), settling down at each interface according to their different size (Figure 1). The cellular material at the two interfaces is entirely recollected with a Pasteur pipette (starting from the upper one and being very careful not to mix the phases while drawing the interfaces) and gently poured into a clean tube. Ice-cold HBSS-FBS is quickly added to the sample up to a final volume of 14–15 mL to dilute the Lympholyte and minimize cellular stress. The suspension is inverted up and down a couple of times and centrifuged at 1800 rpm for 5 min at +4 °C; the islet pellet is finally resuspended in 1 mL of DMEM(−)-BCS (DMEM without glucose, 10% BCS (Bovine Calf Serum), 1% P/S, 0.34% L-Glutamine, 0.1% Gentamicin, 1% Amphotericin B) supplemented with glucose 3.3 mM, then plated in a 24-well (Figure 2) and put in a humidified incubator at 37 °C + 5% CO_2_ from 30 min to overnight, to promote cell recovery.

**Figure 3:**
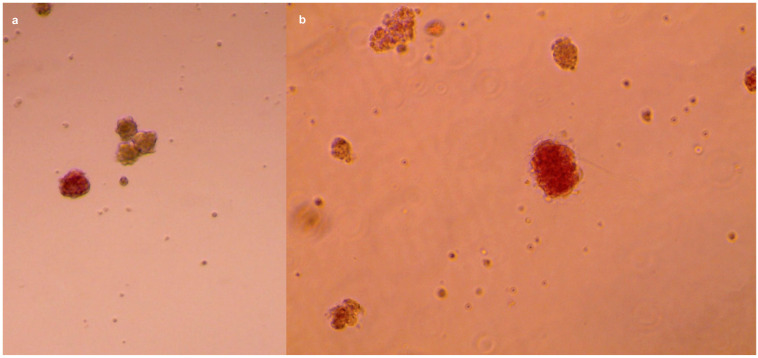
Dithizone staining confirming the presence of pancreatic islets (in purple). The images were acquired through EVOS™ XL Core Imaging System (#AMEX1000): (**a**) captured at a 10× magnification; (**b**) 20× enlargement.

A quick staining with 100 μg/mL dithizone (1:100 from a 10 mg/mL stock) [47] confirms the presence of pancreatic islets (Figure 3).

### 2.5. Glucose Stimulation and Insulin Secretion

#### 2.5.1. Islets Stimulation

To confirm the presence of a physiological β-cell response (i.e., glucose-induced insulin release), the islets were exposed, after the recovery period, to different glucose concentrations. The cells were first transferred in a 1.5 mL tube, gently resuspended, and centrifuged at 1800 rpm for 5 min at +4 °C using a pre-chilled bench centrifuge. The supernatant was fully discarded (without disturbing the cell pellet) to remove the insulin released into the medium during the recovery time. The islet pellet was resuspended in DMEM-BCS and divided equally into three 1.5 mL tubes, distributing 150 μL for each tube. Normalization was achieved by using consistently comparable samples in terms of size: this principle was applied by roughly estimating the islet pellet dimensions and adjusting accordingly the amount of pellet to be assayed. Glucose and arginine were added to the cell suspensions from previously reconstituted stocks (glucose = 334 mM, 1:100 or 1:20; arginine = 400 mM, 1:20) to reach the following concentrations in a final volume of 200 μL/sample:

- Basal glucose, 3.3 mM;
- High glucose, 16.7 mM;
- Basal glucose 3.3 mM+ arginine 20 mM (referred to as arginine 20 mM).

To prepare stocks of glucose and arginine, the powders were resuspended in DMEM(−)-BCS, filtered (0.22 μM), and stored at +4 °C.

Cells were stimulated at 37 °C + 5% CO_2_ for one hour. The samples were then centrifuged at 1800–2000 rpm × 5 min at +4 °C, and the supernatants were separately collected on ice and immediately stored at −80 °C.

#### 2.5.2. Assessment of Insulin and Proinsulin Secretion

Supernatants from non-diabetic (ND)-, pre-diabetic (IGT)-, and diabetic (DM)-derived islets were thawed on ice, and human insulin and proinsulin were measured by commercial ELISA kits, following the manufacturer’s recommendations. Insulin and proinsulin content was reported as OD (optical density) values; stimulation index (SI) for insulin and proinsulin was calculated as follows:

SI (Glucose 16.7 mM) = (OD insulin (or proinsulin) at glucose 16.7 mM)/(OD insulin (or proinsulin) at glucose 3.3 mM);
SI (Arginine 20 mM) = (OD insulin (or proinsulin) at arginine 20 mM)/(OD insulin (or proinsulin) at glucose 3.3 mM);
Mean differences among the three groups were analyzed by one-way analysis of variance (ANOVA), followed by Tukey’s HSD multiple comparison test.

**Figure 4:**
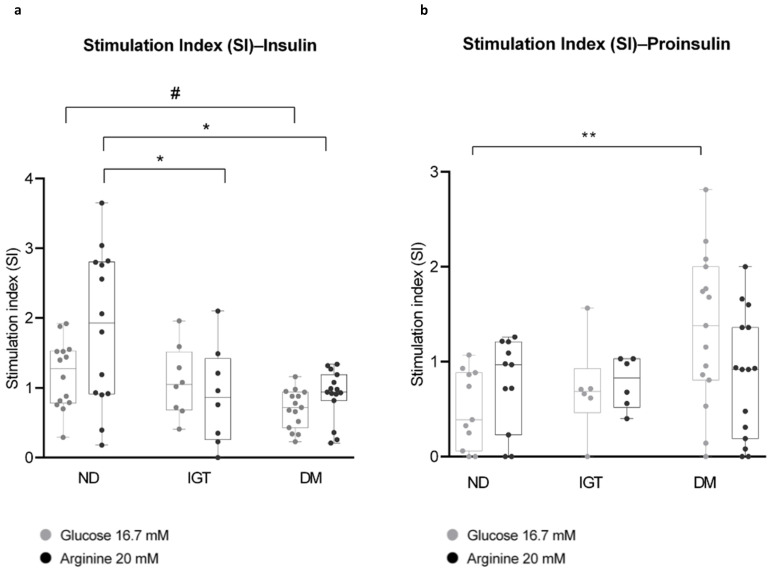
Insulin and proinsulin stimulation index (SI) in islet preparations from non-diabetic (NGT), impaired glucose tolerant (IGT), and diabetic (DM) patients (**a**,**b**). Islets were exposed in vitro to high glucose (Glucose 16.7 mM, in white) or basal glucose + arginine (Arginine 20 mM, in grey); insulin (panel **a**) and proinsulin (Panel **b**) released within the medium were assessed through an ELISA assay. Note that insulin SI is significantly lower in IGT and DM islets compared to ND, especially after arginine stimulation. Conversely, proinsulin levels tend to increase from ND to DM, with a significant increment in the DM group in response to high glucose. One-way ANOVA was applied for the statistics (# *p* < 0.01; * *p* < 0.05 in (**a**); ** *p* < 0.01 in (**b**)), followed by Tukey’s HSD multiple comparison test. All data are presented as mean and ‘min to max’ bars. Human insulin in DMEM-BCS alone was undetectable.

As shown in Figure 4a, insulin SI decreases significantly in DM islets versus ND islets in response to both high glucose and arginine 20 mM and in IGT compared to ND islets for stimulation with arginine 20 mM.

Proinsulin SI, conversely, displayed an increasing trend from ND- to DM-derived islets (with a significant difference between ND and DM), especially under high glucose stimulation (Figure 4b).

To estimate cell death in parallel with insulin and proinsulin secretion, we assayed LDH enzyme activity released in the islets supernatants at all the timepoints. LDH assay (G-Biosciences, St. Louis, MO, USA; #786-324) was performed on frozen islet supernatants, following the manufacturer’s instructions, using the internal positive control of the kit (“LDH positive control” diluted 1:10,000 in PBS-1% BSA), while the stimulation medium alone was used as a negative control (or blank). Since our primary aim was not to assess the percentage of viable cells through this assay, we quantified only secreted LDH within the supernatants, not total LDH. LDH detection was well below the threshold of the positive control (dotted line, CTRL 100% death) for all three groups, thus reasonably ensuring our samples’ acceptable levels of viability (Supplementary Figure S2A). Furthermore, we investigated any possible correlation between LDH values and SI (Supplementary Figure S2B); however, none of the linear regressions proved statistically significant, highlighting the apparent independence of the variables considered.

We also investigated whether a single centrifugation step after filtration, before density gradient separation, was enough to ensure islet enrichment in the final sample (refer to the procedure described in Section 2.3). To address this question, the supernatant that would otherwise be discarded from two distinct samples underwent serial centrifugations. Subsequently, two separate glucose stimulation assays were conducted on the resulting pellets, following the previously described protocol. Overall, insulin levels were found to be exceedingly low and scarcely detectable within the supernatants, thus confirming that viable islets are mostly pulled down during the first centrifugation step after filtration.

Finally, we performed serial islet stimulation assays (i.e., the same batch of islets have been challenged at high glucose following basal glucose stimulation) in a new set of samples. Preliminary data on a small sample set (ND = 3; DM = 3) show a clear trend of increased baseline (3 mM glucose) insulin secretion in non-diabetics compared to DM and a response to high glucose + arginine, which is not observed in diabetic subjects (Figure S3).

Thus, collectively, the data from stimulation experiments confirm that (a) islet preparations are competent for beta cell response to physiological stimuli, and (b) their responses in vitro reflect the metabolic and glycemic profile of the donor as assessed in the clinical setting.

## 3. Discussion

Pancreatic islet isolation plays a crucial role in diabetes research by providing a controlled environment to study β-cell function, investigate disease mechanisms, test potential treatments, and develop therapeutic strategies. Optimizing the whole procedure can be challenging due to the endless variants that can affect the outcome of the protocol and the final product yield and quality. These include the clinical state of the donor and the conditions of tissue preservation [48]; the source of the specimen, since islets are apparently differently distributed in humans between head, body, and tail [49]; and also the enzymes used for digestion [50]. Of note, the clinical and family history of brain-dead organ donors is very limited, and within the specific context of diabetes research, a deep assessment of the patients’ glycemic profiles is impossible, as is the post-surgery follow-up of their metabolic state [5].

On the other hand, several studies have been conducted on pancreatic tissue slices [38,51,52,53,54] and also on purified islets derived from surgical specimens or pancreatectomized patients, generally referred to as living donors [12,55,56,57,58]. The greatest advantage of this model is that it allows for extensive metabolic characterization of the patient before and potentially even after the surgery; a series of clinical and β-cell functional parameters can be assessed or inferred through different kinds of tests (OGTT, euglycemic/hyperglycemic clamp, mixed meal test), having, in the end, a comprehensive patient-specific overlook of the endocrine compartment functionality [37].

In this work, we describe an improved protocol for the isolation of the islets from living, partially pancreatectomized donors. The procedure has been optimized for a minimal amount of starting material (about 1.5 g of pancreatic tissue); while this quantity is similar to what described by Bötticher et al. [12], their reported sample weight range (2–15 g) is still moderately larger than ours, which features our starting conditions as particularly challenging. Indeed, the initial amount of tissue and the histological features of the sample have a great impact on the outcome of the whole process since the final yield can be highly variable depending both on the non-cancerous tissue available from the surgery and on the overall disease status of the patient. As an example, it is known that extensive fibrosis and inflammation are a hallmark of pancreata in subjects affected by chronic pancreatitis [59], but they can also occur in the exocrine compartment of T2D (Type 2 Diabetes) patients [60]. For our purposes, an altered or disrupted tissue morphology represents a fundamental issue to be taken into account during the islets purification process due to the fact that an inflammatory milieu negatively affects islets functionality [61] and also because the increased stiffness of fibrotic tissue [62] could critically impair the efficiency of the digestion step. All these variables, together with a series of technical issues and the nature of reagents used (FBS/BCS of animal derivation, Lympholyte^®^ specifically designed for research purposes), contribute to making this specific protocol unsuitable for clinical and translational applications (e.g., islets transplantation). Of note, isolation protocols aimed at auto- or allotransplantation importantly require a tissue digestion step performed through pancreatic perfusion/intraductal cannulation to ensure the highest possible release of islets from parenchyma; moreover, the whole procedure must be strictly conducted according to current good tissue practice (cGTP) or current good manufacturing practice (cGMP) protocols, and adequate standards of number and purity must be reached for the graft to be safe and effective [63,64]. An upgrade to GMP standards could indeed represent an important future development of the procedure described here.

Functionally, our islets display a physiological insulin response to low/high glucose (which mimics in vitro the physiological glycemic fluctuations occurring within the human body) and to arginine, a powerful secretagogue that elicits a massive release of insulin β-cells granules. Moreover, the insulin secretory response of β-cells—calculated in terms of SI at higher stimuli over basal glycemic conditions—is differentially regulated among the three study groups, particularly during arginine administration. This implies that the purified islets are not only viable and functional but also recapitulate in vitro the metabolic and endocrine features profiled within their respective donors at a clinical level.

Moreover, the analysis of proinsulin SI shows an inverse trend with respect to insulin, i.e., it is higher in the DM group compared to ND. Proinsulin is physiologically secreted at lower concentrations than insulin [65], and increased circulating proinsulin levels have been reported to be higher in T2D patients [66,67]; therefore, this condition has been considered a marker of β-cell dysfunction in diabetic and pre-diabetic subjects [68,69]. Our results are perfectly aligned with these earlier findings, although more samples are needed to validate the significance of the analysis. On the other hand, our method does not detect changes in glucose-stimulated proinsulin secretion in islets from IGT patients. While this limitation may depend on insufficient sampling, the results could also reveal a better-preserved islet function in this patient subgroup compared to DM. Interestingly, we could not detect impaired GSIS in IGT individuals either (Figure 4a), suggesting that peripheral insulin resistance may predominate in these dysmetabolic subjects, although this aspect deserves further investigation.

As discussed above, one of the main challenges of our method is the high variability of the experimental outcome, partially depending on the amount and quality of each specimen. The most relevant source of variability, however, relies on the inter-individual islet heterogeneity involving both genetic and epigenetic features. These features reflect the unique α- and β-functional asset of each donor, as well as the intra-islet diversity within each subject, defined by different endocrine subpopulations, β-cell hubs, and different insulin-secretion rates according to islet size and IR signaling [5,42,70,71,72,73,74,75]. Therefore, it is likely that not all the islets from the same sample equally respond to glucose. In our specific case, since a relatively limited number of islets was randomly distributed in three tubes for stimulation, an uneven proportion of responsive and non-responsive islets may have contributed to an increase in the overall variability of the insulin SI for each sample. For these reasons, it can be even more challenging to obtain consistent results from functional evaluations performed on a reduced-sized sample, which we assume to be representative of the whole pancreatic endocrine complexity. Furthermore, future improvements are needed to standardize the procedure better and control the quality of the samples, e.g., for islet viability (using Fluorescein Diacetate (FDA) or Propidium Iodide (PI)) and purity, before functional analyses.

In conclusion, we believe that the present study has significant novelty compared to the reported publications. In particular, our protocol is applied to extremely low amounts of pancreatic specimens, often less than 2 g (to the authors’ knowledge, the smallest amount used for islets isolation), and does not employ any digestion chamber or cell separator (Ricordi chamber, COBE processor), which makes it much more affordable to most laboratories. In addition, before undergoing surgery, the subjects recruited in this study performed a deep metabolic evaluation, including OGTT, MMT, hyperinsulinemic–euglycemic clamp, and hyperglycemic clamp. This metabolic evaluation and the additional mathematical modeling on the C-peptide curve allow for determining specific β-cell functional parameters. Further, these results can be related to results obtained from ex vivo functional experiments and can allow for defining the changes in secretory pattern ex vivo in different metabolic conditions, ranging from normal glucose tolerance to type 2 diabetic subjects. Such deep in vivo characterization has not been reported in other islet isolation studies, especially from brain-dead donors.

Notwithstanding the above limitations, our results highlight the potential of our method and its broad applications in a future perspective: having a patient-specific model of islets/β-cellular function may represent a breakthrough in T2D research, firstly because the functional state of the endocrine compartment (or at least of part of it) can be characterized in a fine-tuned setting, making it possible to match the secretion data with the subject’s metabolic profile obtained prior surgery and possibly predict the metabolic trajectory of the donor over time. In the second instance, integrating the functional data with the molecular signature disclosed by the -omics approaches may help unravel the complexity of the molecular and cellular basis of T2D. Finally, this kind of model could be of great importance in the drug testing field, particularly in view of optimizing new patient-tailored therapies or validating the existing ones.

## 4. Materials (List of Reagents)

- HBSS (Hank’s Balanced Salts Solution w/Calcium w/Magnesium w/Phenol Red; Euroclone, Pero (MI), Italy, Cat. #ECB4006L);
- 96-Well, Cell Culture-Treated (#353072, Falcon^®^, Corning, NY, USA);
- 24-Well, Cell Culture-Treated (#3524, Corning^®^, Corning, NY, USA);
- Fetal Bovine Serum (Merck-Millipore–Sigma-Aldrich, Darmstadt, Germany, Cat. #F7524);
- Collagenase P, 1.5 U/mg (Merck-Millipore–Sigma-Aldrich, Darmstadt, Germany, Cat. #11249002001);
- Lympholyte^®^-H, sterile liquid (Euroclone, Pero (MI), Italy, Cat. #DVCL5020);
- DMEM, no glucose, no glutamine, no phenol red (Gibco™, Thermo Fisher Scientific Waltham, MA, USA, Cat. #A1443001);
- Bovine Calf Serum (BCS), US Origin (Cytiva, Global Life Sciences Solutions Marlborough, MA, USA, Cat. #SH30073.03);
- L-Arginine Minimum 98% (Merck-Millipore–Sigma-Aldrich, Darmstadt, Germany, Cat. #A-5006);
- D(+)-Glucose Anhydrous (Merck-Millipore–Sigma-Aldrich, Darmstadt, Germany, Cat. #G-5767);
- Amphotericin B (Fungizone→) 250 μg/mL (100 mL) (Euroclone, Pero (MI), Italy, Cat. #ECM0009D);
- Penicillin–Streptomycin 10,000 U-10 mg (Merck-Millipore–Sigma-Aldrich, Darmstadt, Germany, Cat. #P0781);
- L-Glutamine Solution 200 mM (Merck-Millipore–Sigma-Aldrich, Darmstadt, Germany);
- HEPES buffer 1 M (Eurobio Scientific, Les Ulis, France, Cat. #CSTHEP00-0U);
- Gentamicin solution 50 mg/mL (Merck-Millipore–Sigma-Aldrich, Darmstadt, Germany, Cat. #G1397);
- Tweezers;
- Single-use stainless surgical blades (Paragon Medical, Pierceton, IN, USA, Cat. #P301);
- Polypropylene 15 mL–50 mL Graduated Tubes (Sarstedt, Nümbrecht, Germany, Cat. #62 554502, #62 547254);
- Petri dish, 60 × 15 mm, transparent, with ventilation cams (Sarstedt, Nümbrecht, Germany, Cat. #82.1194.500);
- Corning^®^ Cell strainer, pore size 100 μm and 40 μm (Cat. #431752, #431750);
- Pipettes and tips (2–100 μL);
- MACROMAN Pipette controller (Gilson, Middleton, WI, USA, Cat. #F110120), serological pipettes;
- Shaking water bath;
- SL 16 Centrifuge Series (Thermo Scientific™, Thermo Fisher Scientific, Waltham, MA, USA, Cat. #75004031);
- Series 8000 Direct-Heat CO_2_ Incubator (Thermo Scientific™, Thermo Fisher Scientific Waltham, MA, USA, Cat. #3540-MAR);
- Ethanol 70%;
- 3.5 mL Transferpipette (Sarstedt, Nümbrecht, Germany, Cat. # 86.1171.001);
- Human Insulin ELISA kit (Merck-Millipore–Sigma-Aldrich, Darmstadt, Germany, #EZHI-14K);
- Human Total Proinsulin ELISA kit (Merck-Millipore–Sigma-Aldrich, Darmstadt, Germany, #EZHPI-15K);
- Dithizone (Merck-Millipore–Sigma-Aldrich, Darmstadt, Germany, Cat. #D5130);
- CytoScan™ LDH Cytotoxicity Assay (G-Biosciences, St. Louis, MO, USA, #786-324);
- GraphPad Prism v8.0 (GraphPad Software, Boston, MA, USA).

### Tips Section

1. Minimize the sample processing time as much as possible, always using ice-cold buffers/media (except for Lympholyte). Prepare HBSS-FBS fresh aliquots before starting.
2. If the tissue is very fatty or fibrotic, discard the unsuitable parts during cutting. This will improve the overall quality of digestion, especially in the case of fat, which tends to form a superficial oily layer that significantly lowers the final yield.
3. If the starting specimen is particularly small in size, repeat the filtration step through the 40 μm cell strainer to minimize the loss of islets, and thoroughly wash the mesh of the strainers with higher volumes of ice-cold HBSS-FBS.
4. Glucose stimulation experiments are performed by dividing the sample equally into three tubes. This requires a homogeneous cell suspension that can be achieved by pipetting 1/3 of the sample volume from the bottom to the top at least 3–4 times and then drawing the desired volume from the center of the suspension.
5. It is uncommon to see the ‘cell ring’ at the lower interface (3 mL), but it is crucial to proceed nonetheless to improve the yield.
6. It is necessary to handle the density gradient with extreme care, as the interfaces are delicate and prone to easy remixing, resulting in material loss. A 3.5 mL transfer pipette can be very helpful in recollecting almost the whole ‘cell ring’ at the interfaces; however, using a p1000 pipette can be more manageable for beginners.

## Figures and Tables

**Table 1:** Clinical and metabolic characteristics of the enrolled patients classified into ND, IGT, and DM according to glucose tolerance before surgery. Data are represented as means ± SEM (* *p* < 0.05).

Subject Characteristics	ND (*n* = 14)	IGT (*n* = 8)	DM (*n* = 15)	*p* Value
Mean age (y)	58.1 ± 4.02	66 ± 4.38	73.4 ± 2.04	
Gender (F/M)	7/7	3/5	9/6	
BMI (kg/m^2^)	24.67 ± 1.56	25.04 ± 1.55	23.70 ± 0.94	0.77
Insulin sensitivity (mg·kg^−1^·min^−1^)	4.60 ± 1.36	3.20 ± 0.48	4.90 ± 1.37	0.64
Fasting glucose (mg/dL)	86.5 ± 2.29	95.6 ± 5.27	129.4 ± 12.63	0.003 *
Fasting insulin (µUI/mL)	4 ± 0.40	4.9 ± 0.53	4.92 ± 1.11	0.358
Fasting C-peptide (ng/mL)	1.54 ± 0.29	1.13 ± 0.10	2.05 ± 0.29	0.87
AUC insulin (µUI/mL)	11,351.1 ± 1513.6	12,921.5 ± 1730.4	5433 ± 1643	0.0129 *
AUC C-peptide (ng/mL)	1159.9 ± 107.8	1343.1 ± 112.5	703,7 ± 114.7	0.0036 *

## Data Availability

Data is contained within the article and Supplementary Materials.

## Supplementary Materials

The following supporting information can be downloaded at: https://www.mdpi.com/article/10.3390/ijms25115936/s1.

## References

[1] Wesolowska-Andersen A., Brorsson C.A., Bizzotto R., Mari A., Tura A., Koivula R., Mahajan A., Vinuela A., Tajes J.F., Sharma S. (2022). Four groups of type 2 diabetes contribute to the etiological and clinical heterogeneity in newly diagnosed individuals: An IMI DIRECT study. Cell Rep. Med..

[2] Camunas-Soler J., Dai X.Q., Hang Y., Bautista A., Lyon J., Suzuki K., Kim S.K., Quake S.R., MacDonald P.E. (2020). Patch-Seq Links Single-Cell Transcriptomes to Human Islet Dysfunction in Diabetes. Cell Metab..

[3] Segerstolpe Å., Palasantza A., Eliasson P., Andersson E.M., Andréasson A.C., Sun X., Picelli S., Sabirsh A., Clausen M., Bjursell M.K. (2016). Single-Cell Transcriptome Profiling of Human Pancreatic Islets in Health and Type 2 Diabetes. Cell Metab..

[4] Avrahami D., Wang Y.J., Schug J., Feleke E., Gao L., Liu C., Naji A., Glaser B., Kaestner K.H., HPAP Consortium (2020). Single-cell transcriptomics of human islet ontogeny defines the molecular basis of β-cell dedifferentiation in T2D. Mol. Metab..

[5] Gloyn A.L., Ibberson M., Marchetti P., Powers A.C., Rorsman P., Sander M., Solimena M. (2022). Every islet matters: Improving the impact of human islet research. Nat. Metab..

[6] Shapiro A.M.J., Pokrywczynska M., Ricordi C. (2017). Clinical pancreatic islet transplantation. Nat. Rev. Endocrinol..

[7] Halberstadt C., Williams D., Gores P. (2013). Isolation of human cadaveric pancreatic islets for clinical transplantation. Methods Mol. Biol..

[8] De Paep D.L., Van Hulle F., Ling Z., Vanhoeij M., Hilbrands R., Distelmans W., Gillard P., Keymeulen B., Pipeleers D., Jacobs-Tulleneers-Thevissen D. (2022). Utility of Islet Cell Preparations from Donor Pancreases After Euthanasia. Cell Transplant..

[9] Ricordi C., Lacy P.E., Finke E.H., Olack B.J., Scharp D.W. (1988). Automated method for isolation of human pancreatic islets. Diabetes.

[10] Ricordi C., Lacy P.E., Scharp D.W. (1989). Automated Islet Isolation from Human Pancreas. Diabetes.

[11] Wei L., Moore A., Wang P. (2023). Isolation and Purification of Human Pancreatic Islets. Type-1 Diabetes.

[12] Bötticher G., Sturm D., Ehehalt F., Knoch K.P., Kersting S., Grützmann R., Baretton G.B., Solimena M., Saeger H.D. (2011). Isolation of human islets from partially pancreatectomized patients. J. Vis. Exp..

[13] Qi M., Barbaro B., Wang S., Wang Y., Hansen M., Oberholzer J. (2009). Human pancreatic islet isolation: Part II: Purification and culture of human islets. J. Vis. Exp..

[14] Töns H.A.M., Baranski A.G., Terpstra O.T., Bouwman E. (2008). Isolation of the islets of Langerhans from the human pancreas with magnetic retraction. Transplant. Proc..

[15] Miki A., Ricordi C., Messinger S., Yamamoto T., Mita A., Barker S., Haetter R., Khan A., Alejandro R., Ichii H. (2009). Toward improving human islet isolation from younger donors: Rescue purification is efficient for trapped islets. Cell Transplant..

[16] Saliba Y., Farès N. (2019). Isolation, Purification, and Culture of Mouse Pancreatic Islets of Langerhans. Methods Mol. Biol..

[17] O’Dowd J.F., Stocker C.J. (2020). Isolation and Purification of Rodent Pancreatic Islets of Langerhans. Methods Mol. Biol..

[18] Li D.S., Yuan Y.H., Tu H.J., Liang Q.L., Dai L.J. (2009). A protocol for islet isolation from mouse pancreas. Nat. Protoc..

[19] Corbin K.L., West H.L., Brodsky S., Whitticar N.B., Koch W.J., Nunemaker C.S. (2021). A Practical Guide to Rodent Islet Isolation and Assessment Revisited. Biol. Proced. Online.

[20] Heiser A., Ulrichs K., Müller-Ruchholtz W. (1994). Isolation of porcine pancreatic islets: Low trypsin activity during the isolation procedure guarantees reproducible high islet yields. J. Clin. Lab. Anal..

[21] Krickhahn M., Bühler C., Meyer T., Thiede A., Ulrichs K. (2002). The morphology of islets within the porcine donor pancreas determines the isolation result: Successful isolation of pancreatic islets can now be achieved from young market pigs. Cell Transplant..

[22] Brandhorst H., Johnson P.R.V., Brandhorst D. (2016). Pancreatic Islets: Methods for Isolation and Purification of Juvenile and Adult Pig Islets. Adv. Exp. Med. Biol..

[23] Lu Y., Pu Z., Chen J., Deng J., Deng Y., Zhu S., Xu C., Yao F., Wu Z., Ni Y. (2021). Adult Pig Islet Isolation. J. Vis. Exp..

[24] MacDonald M.J., Longacre M.J., Stoker S.W., Kendrick M., Thonpho A., Brown L.J., Hasan N.M., Jitrapakdee S., Fukao T., Hanson M.S. (2011). Differences between human and rodent pancreatic islets: Low pyruvate carboxylase, atp citrate lyase, and pyruvate carboxylation and high glucose-stimulated acetoacetate in human pancreatic islets. J. Biol. Chem..

[25] Levetan C.S., Pierce S.M. (2013). Distinctions between the islets of mice and men: Implications for new therapies for type 1 and 2 diabetes. Endocr. Pract..

[26] Bellin M.D., Dunn T.B. (2020). Transplant strategies for type 1 diabetes: Whole pancreas, islet and porcine beta cell therapies. Diabetologia.

[27] Dhanasekaran M., George J.J., Loganathan G., Narayanan S., Hughes M.G., Williams S.K., Balamurugan A.N. (2017). Pig islet xenotransplantation. Curr. Opin. Organ. Transplant..

[28] Muraro M.J., Dharmadhikari G., Grün D., Groen N., Dielen T., Jansen E., van Gurp L., Engelse M.A., Carlotti F., de Koning E.J. (2016). A Single-Cell Transcriptome Atlas of the Human Pancreas. Cell Syst..

[29] Li J., Klughammer J., Farlik M., Penz T., Spittler A., Barbieux C., Berishvili E., Bock C., Kubicek S. (2016). Single-cell transcriptomes reveal characteristic features of human pancreatic islet cell types. EMBO Rep..

[30] Ciregia F., Bugliani M., Ronci M., Giusti L., Boldrini C., Mazzoni M.R., Mossuto S., Grano F., Cnop M., Marselli L. (2017). Palmitate-induced lipotoxicity alters acetylation of multiple proteins in clonal β cells and human pancreatic islets. Sci. Rep..

[31] Marselli L., Piron A., Suleiman M., Colli M.L., Yi X., Khamis A., Carrat G.R., Rutter G.A., Bugliani M., Giusti L. (2020). Persistent or Transient Human β Cell Dysfunction Induced by Metabolic Stress: Specific Signatures and Shared Gene Expression with Type 2 Diabetes. Cell Rep..

[32] Goto M., Eich T.M., Felldin M., Foss A., Källen R., Salmela K., Tibell A., Tufveson G., Fujimori K., Engkvist M. (2004). Refinement of the automated method for human islet isolation and presentation of a closed system for in vitro islet culture. Transplantation.

[33] Skog O., Korsgren S., Wiberg A., Danielsson A., Edwin B., Buanes T., Krogvold L., Korsgren O., Dahl-Jørgensen K. (2015). Expression of human leukocyte antigen class I in endocrine and exocrine pancreatic tissue at onset of type 1 diabetes. Am. J. Pathol..

[34] Marchetti P., Suleiman M., Marselli L. (2018). Organ donor pancreases for the study of human islet cell histology and pathophysiology: A precious and valuable resource. Diabetologia.

[35] Mezza T., Cefalo C.M.A., Cinti F., Quero G., Pontecorvi A., Alfieri S., Holst J.J., Giaccari A. (2020). Endocrine and Metabolic Insights from Pancreatic Surgery. Trends Endocrinol. Metab..

[36] Mezza T., Clemente G., Sorice G.P., Conte C., De Rose A.M., Sun V.A., Cefalo C.M., Pontecorvi A., Nuzzo G., Giaccari A. (2015). Metabolic consequences of the occlusion of the main pancreatic duct with acrylic glue after pancreaticoduodenectomy. Am. J. Surg..

[37] Mezza T., Ferraro P.M., Di Giuseppe G., Moffa S., Cefalo C.M., Cinti F., Impronta F., Capece U., Quero G., Pontecorvi A. (2021). Pancreaticoduodenectomy model demonstrates a fundamental role of dysfunctional β cells in predicting diabetes. J. Clin. Investig..

[38] Mezza T., Sorice G.P., Conte C., Sun V.A., Cefalo C.M., Moffa S., Pontecorvi A., Mari A., Kulkarni R.N., Giaccari A. (2016). β-Cell Glucose Sensitivity Is Linked to Insulin/Glucagon Bihormonal Cells in Nondiabetic Humans. J. Clin. Endocrinol. Metab..

[39] DeFronzo R.A., Ferrannini E., Groop L., Henry R.R., Herman W.H., Holst J.J., Hu F.B., Kahn C.R., Raz I., Shulman G.I. (2015). Type 2 diabetes mellitus. Nat. Rev. Dis. Primers.

[40] Berkova Z., Saudek F., Girman P., Zacharovova K., Kriz J., Fabryova E., Leontovyc I., Koblas T., Kosinova L., Neskudla T. (2016). Combining Donor Characteristics with Immunohistological Data Improves the Prediction of Islet Isolation Success. J. Diabetes Res..

[41] Komatsu H., Cook C., Wang C.H., Medrano L., Lin H., Kandeel F., Tai Y.C., Mullen Y. (2017). Oxygen environment and islet size are the primary limiting factors of isolated pancreatic islet survival. PLoS ONE.

[42] Dybala M.P., Hara M. (2019). Heterogeneity of the Human Pancreatic Islet. Diabetes.

[43] Huang H.H., Harrington S., Stehno-Bittel L. (2018). The Flaws and Future of Islet Volume Measurements. Cell Transplant..

[44] Spinelli E., Caporale R., Buchi F., Masala E., Gozzini A., Sanna A., Sassolini F., Valencia A., Bosi A., Santini V. (2012). Distinct signal transduction abnormalities and erythropoietin response in bone marrow hematopoietic cell subpopulations of myelodysplastic syndrome patients. Clin. Cancer Res..

[45] Colasanti T., Alessandri C., Capozzi A., Sorice M., Delunardo F., Longo A., Pierdominici M., Conti F., Truglia S., Siracusano A. (2012). Autoantibodies specific to a peptide of β2-glycoprotein I cross-react with TLR4, inducing a proinflammatory phenotype in endothelial cells and monocytes. Blood.

[46] Pievani A., Belussi C., Klein C., Rambaldi A., Golay J., Introna M. (2011). Enhanced killing of human B-cell lymphoma targets by combined use of cytokine-induced killer cell (CIK) cultures and anti-CD20 antibodies. Blood.

[47] NIH CIT Consortium Chemistry Manufacturing Controls Monitoring Committee, NIH CIT Consortium (2015). Purified Human Pancreatic Islet: Qualitative and Quantitative Assessment of Islets Using Dithizone (DTZ): Standard Operating Procedure of the NIH Clinical Islet Transplantation Consortium. CellR4 Cell. Repair Replace. Regen. Reprogramming.

[48] Kaddis J.S., Danobeitia J.S., Niland J.C., Stiller T., Fernandez L.A. (2010). Multicenter analysis of novel and established variables associated with successful human islet isolation outcomes. Am. J. Transplant..

[49] Wang X., Misawa R., Zielinski M.C., Cowen P., Jo J., Periwal V., Ricordi C., Khan A., Szust J., Shen J. (2013). Regional differences in islet distribution in the human pancreas—Preferential beta-cell loss in the head region in patients with type 2 diabetes. PLoS ONE.

[50] Barnett M.J., Zhai X., LeGatt D.F., Cheng S.B., Shapiro A.M.J., Lakey J.R.T. (2005). Quantitative assessment of collagenase blends for human islet isolation. Transplantation.

[51] Yoon K.H., Ko S.H., Cho J.H., Lee J.M., Ahn Y.B., Song K.H., Yoo S.J., Kang M.I., Cha B.Y., Lee K.W. (2003). Selective beta-cell loss and alpha-cell expansion in patients with type 2 diabetes mellitus in Korea. J. Clin. Endocrinol. Metab..

[52] Meier J.J., Breuer T.G.K., Bonadonna R.C., Tannapfel A., Uhl W., Schmidt W.E., Schrader H., Menge B.A. (2012). Pancreatic diabetes manifests when beta cell area declines by approximately 65% in humans. Diabetologia.

[53] Cohrs C.M., Chen C., Jahn S.R., Stertmann J., Chmelova H., Weitz J., Bähr A., Klymiuk N., Steffen A., Ludwig B. (2017). Vessel Network Architecture of Adult Human Islets Promotes Distinct Cell-Cell Interactions In Situ and Is Altered after Transplantation. Endocrinology.

[54] Yoneda S., Uno S., Iwahashi H., Fujita Y., Yoshikawa A., Kozawa J., Okita K., Takiuchi D., Eguchi H., Nagano H. (2013). Predominance of β-Cell Neogenesis Rather Than Replication in Humans with an Impaired Glucose Tolerance and Newly Diagnosed Diabetes. J. Clin. Endocrinol. Metab..

[55] Matsumoto S., Okitsu T., Iwanaga Y., Noguchi H., Nagata H., Yonekawa Y., Yamada Y., Nakai Y., Ueda M., Ishii A. (2005). Insulin independence of unstable diabetic patient after single living donor islet transplantation. Transplant. Proc..

[56] Oh J.Y., Kim Y.H., Lee S., Lee Y.N., Go H.S., Hwang D.W., Song K.B., Lee J.H., Lee W., So S. (2022). The Outcomes and Quality of Pancreatic Islet Cells Isolated from Surgical Specimens for Research on Diabetes Mellitus. Cells.

[57] Krogvold L., Wiberg A., Edwin B., Buanes T., Jahnsen F.L., Hanssen K.F., Larsson E., Korsgren O., Skog O., Dahl-Jørgensen K. (2016). Insulitis and characterisation of infiltrating T cells in surgical pancreatic tail resections from patients at onset of type 1 diabetes. Diabetologia.

[58] Ehehalt F., Knoch K., Erdmann K., Krautz C., Jäger M., Steffen A., Wegbrod C., Meisterfeld R., Kersting S., Bergert H. (2010). Impaired insulin turnover in islets from type 2 diabetic patients. Islets.

[59] Braganza J.M., Lee S.H., McCloy R.F., McMahon M.J. (2011). Chronic pancreatitis. Lancet.

[60] Wright J.J., Eskaros A., Windon A.L., Saunders D.C., Bottino R., Brissova M., Powers A.C., Human Pancreas Analysis Program (2022). 1374-P: In Type 2 Diabetes, the Exocrine Pancreas Has Greater Fibrosis, Fat, Metaplastic Changes, and Microangiopathy. Diabetes.

[61] Collier J.J., Sparer T.E., Karlstad M.D., Burke S.J. (2017). Pancreatic islet inflammation: An emerging role for chemokines. J. Mol. Endocrinol..

[62] Ferdek P.E., Krzysztofik D., Stopa K.B., Kusiak A.A., Paw M., Wnuk D., Jakubowska M.A. (2022). When healing turns into killing—The pathophysiology of pancreatic and hepatic fibrosis. J. Physiol..

[63] Rickels M.R., Robertson R.P. (2019). Pancreatic Islet Transplantation in Humans: Recent Progress and Future Directions. Endocr. Rev..

[64] Cayabyab F., Nih L.R., Yoshihara E. (2021). Advances in Pancreatic Islet Transplantation Sites for the Treatment of Diabetes. Front. Endocrinol..

[65] Henquin J.C. (2021). Glucose-induced insulin secretion in isolated human islets: Does it truly reflect β-cell function in vivo?. Mol. Metab..

[66] Porte D. (1991). Banting lecture 1990. Beta-cells in type II diabetes mellitus. Diabetes.

[67] Ward W.K., Bolgiano D.C., McKnight B., Halter J.B., Porte D. (1984). Diminished B cell secretory capacity in patients with noninsulin-dependent diabetes mellitus. J. Clin. Investig..

[68] Gorden P., Hendricks C.M., Roth J. (1974). Circulating proinsulin-like component in man: Increased proportion in hypoinsulinemic states. Diabetologia.

[69] Mezza T., Ferraro P.M., Sun V.A., Moffa S., Cefalo C.M.A., Quero G., Cinti F., Sorice G.P., Pontecorvi A., Folli F. (2018). Increased β-Cell Workload Modulates Proinsulin-to-Insulin Ratio in Humans. Diabetes.

[70] Wigger L., Barovic M., Brunner A.D., Marzetta F., Schöniger E., Mehl F., Kipke N., Friedland D., Burdet F., Kessler C. (2021). Multi-omics profiling of living human pancreatic islet donors reveals heterogeneous beta cell trajectories towards type 2 diabetes. Nat. Metab..

[71] Baron M., Veres A., Wolock S.L., Faust A.L., Gaujoux R., Vetere A., Ryu J.H., Wagner B.K., Shen-Orr S.S., Klein A.M. (2016). A Single-Cell Transcriptomic Map of the Human and Mouse Pancreas Reveals Inter- and Intra-cell Population Structure. Cell Syst..

[72] Johnston N.R., Mitchell R.K., Haythorne E., Pessoa M.P., Semplici F., Ferrer J., Piemonti L., Marchetti P., Bugliani M., Bosco D. (2016). Beta Cell Hubs Dictate Pancreatic Islet Responses to Glucose. Cell Metab..

[73] Fujita Y., Takita M., Shimoda M., Itoh T., Sugimoto K., Noguchi H., Naziruddin B., Levy M.F., Matsumoto S. (2011). Large human islets secrete less insulin per islet equivalent than smaller islets in vitro. Islets.

[74] Brusco N., Sebastiani G., Di Giuseppe G., Licata G., Grieco G.E., Fignani D., Nigi L., Formichi C., Aiello E., Auddino S. (2023). Intra-islet insulin synthesis defects are associated with endoplasmic reticulum stress and loss of beta cell identity in human diabetes. Diabetologia.

[75] Mezza T., Shirakawa J., Martinez R., Hu J., Giaccari A., Kulkarni R.N. (2016). Nuclear Export of FoxO1 Is Associated with ERK Signaling in β-Cells Lacking Insulin Receptors. J. Biol. Chem..

