# Supplementary material for: Advancing Diabetes Research: A Novel Islet Isolation Method from Living Donors
Source: Int J Mol Sci. 2024 May 29;25(11):5936. doi: 10.3390/ijms25115936 (PMC11172646; doi:10.3390/ijms25115936)
Supplement: Supplementary file 1 [file ijms-25-05936-s001.zip › ijms-3024139-supplementary.pdf]

S1

Insulin secretion during hyperglycemic clamp

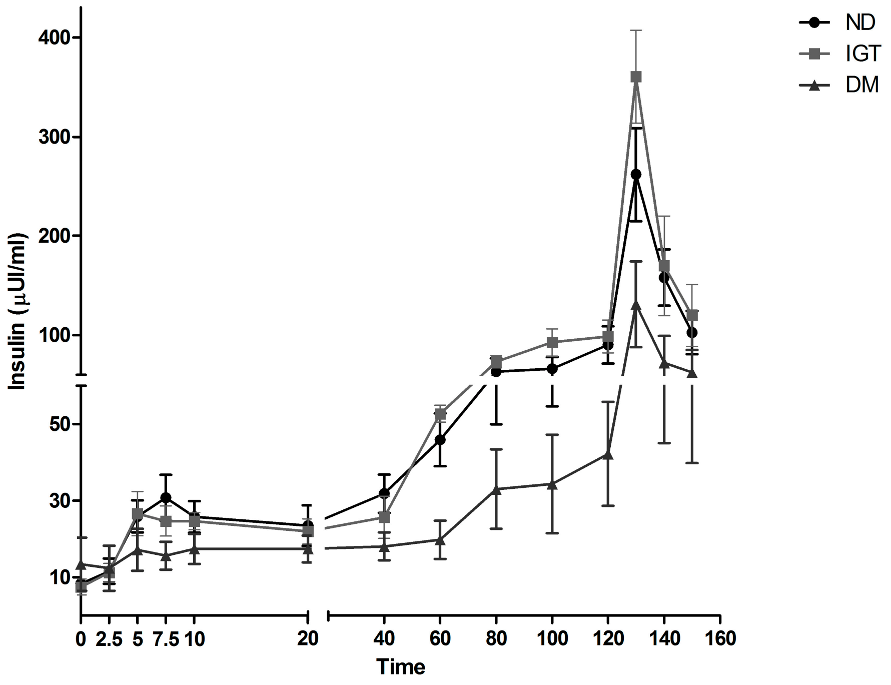

c-peptide secretion during hyperglycemic clamp

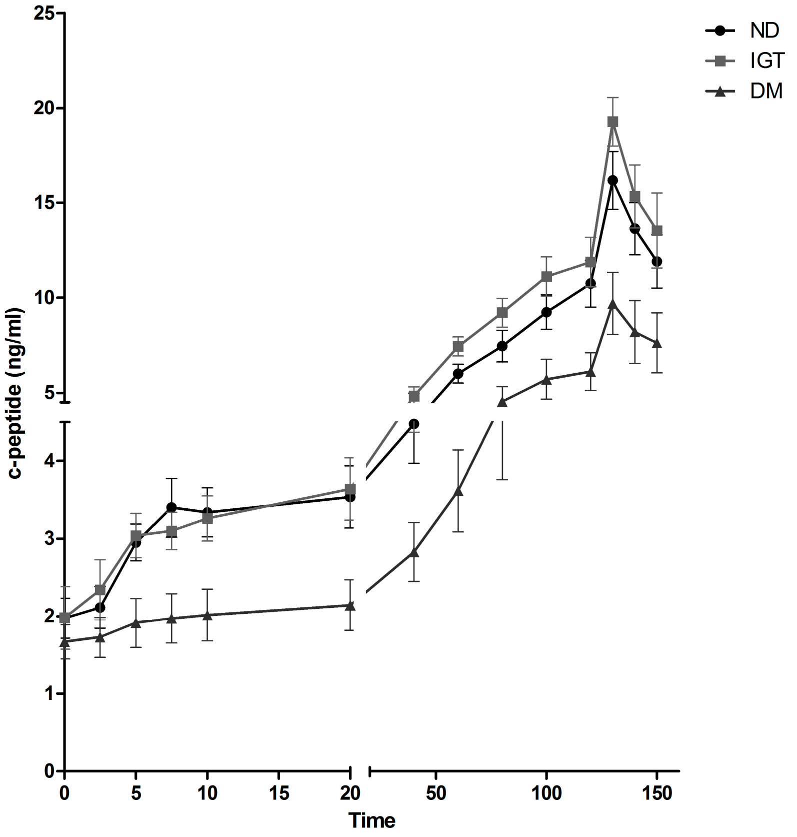

Figure S1: AUC of Insulin and c-peptide secretion monitored during hyperglycemic clamp in ND (n=8), IGT (n=5), DM (n=7) patients.

S2A

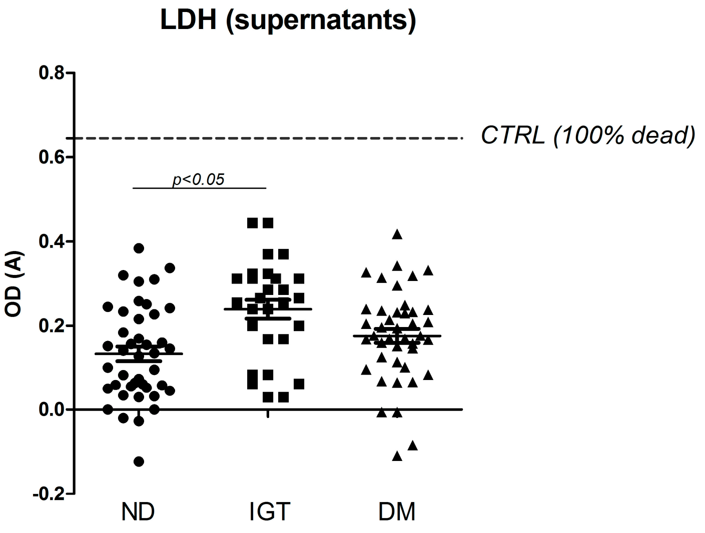

S2B

SI-LDH correlation analysis

ND

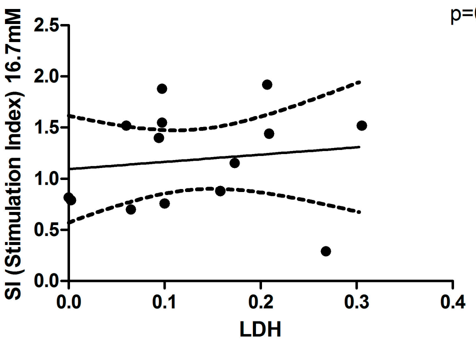

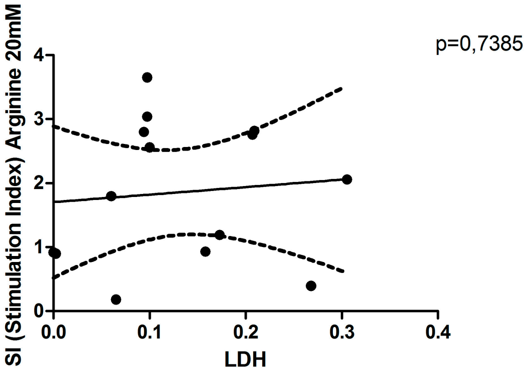

IGT

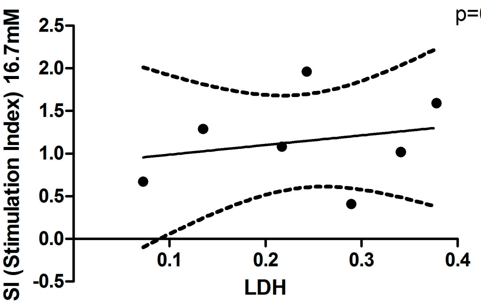

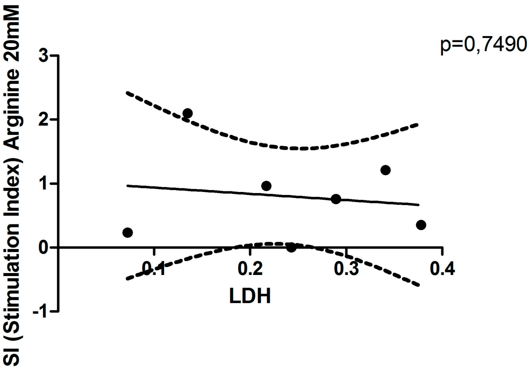

DM

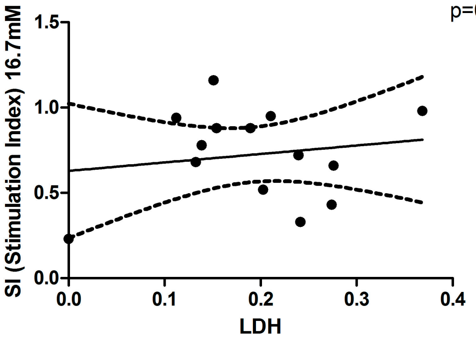

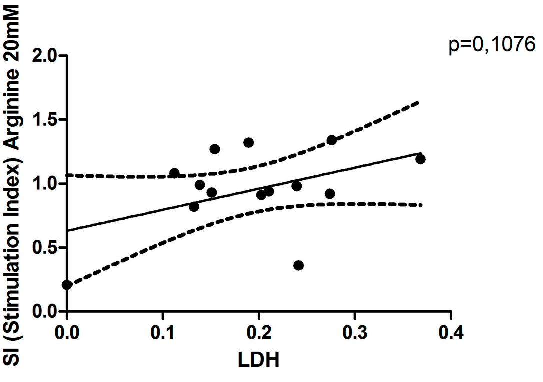

Figure S2: In **S2A**, islets supernatants were assayed for LDH at all timepoints. Despite significantly higher detection in IGT islets compared to ND islets, LDH was well below the threshold of the positive control (100% death) for all three groups. One-way ANOVA was applied for the statistics ($p < 0.05$), followed by Tukey's HSD multiple comparison test. Data are presented as mean +/- SEM for each group. In **S2B**, several linear regression analysis were performed to evaluate any possible correlation between LDH values and SI considering each sample's average LDH value (calculated for 'Glucose 16.7 mM' and 'Arginine 20 mM' supernatants) and its relative SI at each timepoint: neither positive or negative correlations were identified. GraphPad Prism v.8 was used for this analysis.

S3

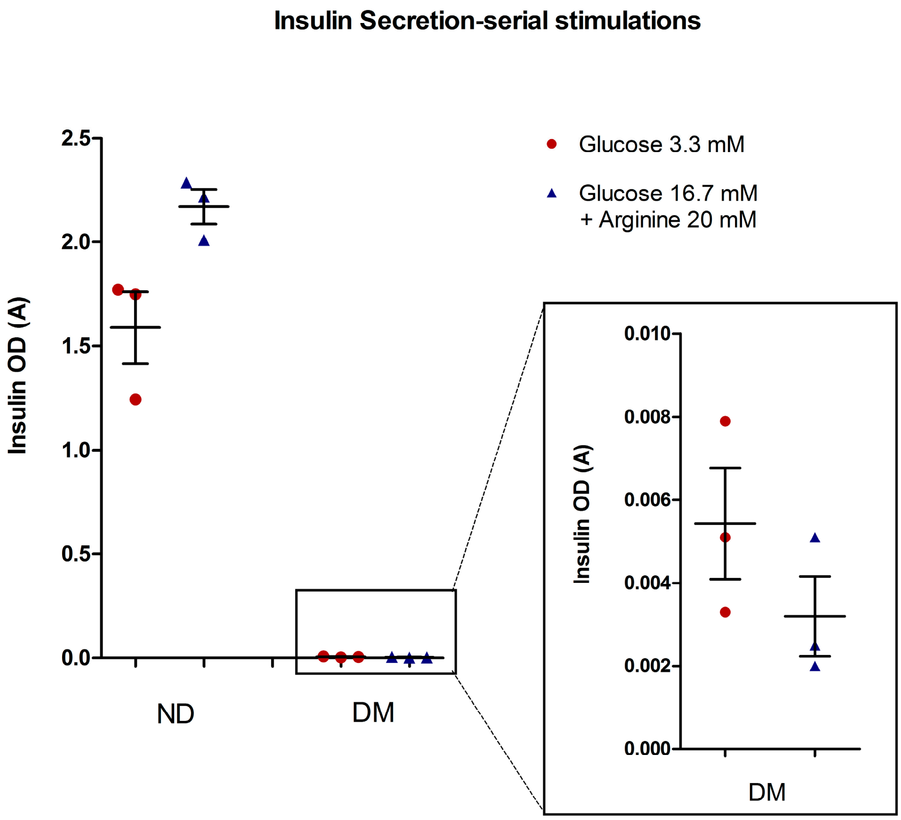

Figure S3: Preliminary data of serial islet stimulation assays (ND: n=3; DM: n=3), in which the same batch of islets have been challenged at high glucose following basal glucose stimulation. The figure shows an increased baseline (3.3 mM glucose) insulin secretion in ND islets and a response to high glucose + arginine in non-diabetics compared to DM. Data are presented as mean +/- SEM.
